# Retention in treatment and therapeutic adherence: How are these associated with therapeutic success? An analysis using real‐world data

**DOI:** 10.1002/mpr.1929

**Published:** 2022-06-28

**Authors:** Daniel Dacosta‐Sánchez, Bella M. González‐Ponce, Fermín Fernández‐Calderón, Manuel Sánchez‐García, Oscar M. Lozano

**Affiliations:** ^1^ Department of Clinical and Experimental Psychology University of Huelva Huelva Spain; ^2^ Research Center on Natural Resources Health and the Environment University of Huelva Huelva Spain

**Keywords:** adherence to treatment, outcomes, retention rates, substance abuse treatment, treatment monitoring

## Abstract

**Introduction:**

Treatment retention and adherence are used as outcomes in numerous randomized clinical trials and observational studies conducted in the addiction field. Although usual criteria are 3/6 months of treatment retention or number of sessions attended, there is not a methodological support for conclusions using these criteria. This study analyzed the usefulness of retention and adherence to predict therapeutic success.

**Methods:**

Retrospective observational study using real‐world data from electronic health records of 11,907 patients in treatment diagnosed with cocaine, alcohol, cannabis and opiate use disorders or harmful use.

**Results:**

Moderate effect size relations were found between the different type of clinical discharge and months in retention (*η* ^2^ = 0.12) and proportion of attendance (*η* ^2^ = 0.10). No relationship was found with the number of sessions attended. Using cut‐off points (i.e., 3 or 6 months in treatment or attending 6 therapy sessions) worsens the ability to predict the type of discharge.

**Discussions/Conclusion:**

Treatment retention and adherence are indicators moderately related to therapeutic success. Research using these indicators to assess the effectiveness of therapies should complement their results with other clinical indicators and quality of life measures.

## 1 INTRODUCTION

Substance Use Disorders (SUD) are considered chronic disorders (Fleury et al., 2016; Lynch et al., 2021) whose treatment usually involves relapse (Sliedrecht et al., 2019), with high rates of non‐attendance at appointments (Milward et al., 2014) and non‐therapeutic discharge (Madoz‐Gúrpide et al., 2013). Thus, this therapeutic course is accompanied by progressive deterioration in the physical, psychological, and social health of patients and their families (Laudet et al., 2009). In addition, treatment dropouts and readmissions are problems that represent a cost overrun for the health systems, harming the management of the centers, and limiting the healthcare provided to patients (Lappan et al., 2020). For this reason, understanding the variables involved in the success of SUD treatment is a line of research of great interest in this field (Acion et al., 2017).

Therapeutic success is determined by a clinical decision based on patients' recovery from abstinence, significant reduction in drug use, as well as other notable improvements in patients' health and quality of life (Lail & Fairbairn, 2018; Piercy et al., 2021). A review of the specialized literature reveals several variables associated with therapeutic success and dropout. These include patient‐related variables such as sociodemographic variables (e.g., age), variables associated with the clinical history and diagnosis of patients (e.g., the pattern of use or the severity of dependence), psychological variables (e.g., motivation or craving) and neuropsychological variables (e.g., inhibitory control or decision making) (Bedard‐Gilligan et al., 2018; Domínguez‐Salas et al., 2016; Reske & Paulus, 2008). In addition, the importance of variables associated with the therapeutic process should also be noted (Simpson, 2001, 2004), including retention and therapeutic adherence, both in terms of compliance with medication guidelines and adherence to appointments (Austin et al., 2015; Steinkamp et al., 2019).

These last two variables have also been shown to be associated with reduced drug use and improvements in patients' quality of life (Lynch et al., 2021; Zhou et al., 2017). For this reason, various addiction treatment programs have among their objectives the promotion of adherence and retention in treatment (Gaulen et al., 2022; Reif et al., 2021), with both aspects of the therapeutic process regarded as dependent variables in research on this issue (Bedard‐Gilligan, 2018; Daigre et al., 2021). Thus, given the use of these two variables in the field of addictions, it is unsurprising that retention and therapeutic adherence have been established as outcomes.

Retention is probably the most widely used indicator of therapeutic outcomes (Fleury et al., 2016; Wiessing et al., 2018), although there is no clear consensus on its definition and its usefulness as an indicator of patient improvemet has been questioned (Dearing et al., 2005; O’Connor et al., 2020; Walker, 2009). Despite this, institutions such as the National Institute on Drug Abuse (NIDA) point out that time in treatment is associated with the effectiveness of intervention programs. Furthermore, this institution claims that most patients need at least 3 months in treatment to observe a significant reduction in drug use (NIDA, 2018), a time period that is supported by other studies (Joe et al., 1999; Simpson, 2001, 2004). This is probably the reason why most of the efficacy studies are carried out with 3‐month follow‐ups. However, it should be noted that this period can vary depending on the severity and characteristics of the addiction, with retention times of between 6 and 24 months being desirable (Hoffman et al., 1996). In this regard, it should be noted that it is common practice to transform retention into a dichotomous variable depending on whether a given period in treatment is achieved (Scheibe et al., 2020; Stahler & Mennis, 2020).

Adherence to appointments has received rather less attention than retention and therapeutic adherence and has been studied mainly among patients in methadone treatment programs (Austin et al., 2015; Viera et al., 2020). However, some studies have indicated that the rates of non‐attendance to appointments range between 10% and 60% (Lefforge et al., 2007), and that these patients present less adherence than the general population (Austin et al., 2015). Moreover, this indicator has been operationalized in various ways. Thus, it has been defined as the number of therapeutic sessions attended by patients, the proportion of appointments attended out of the total number of scheduled appointments, or weekly attendance to a session. As in the case of retention, adherence also appears in the studies as a dichotomous variable in terms of adherence versus non‐adherence, with different cut‐offs (Raes et al., 2011).

Despite the lack of consensus and difficulties in operationalizing retention and therapeutic adherence, the usefulness of these indicators in relation to the clinical course of patients with SUD is unquestionable (Viera et al., 2020). For this reason, some authors point out the need for greater methodological support for the use of these variables and the establishment of categorical thresholds associated with therapeutic success and dropout (Turner & Deane, 2016). In this regard, to complement the previous evidences obtained through ad hoc descriptive studies, it may be useful to use real‐world data, operationalized as a set of data collected by clinicians during clinical practice, which can be obtained from electronic health records (EHR). Some authors have pointed out the potential of using EHRs in the field of addictions (Marsch et al., 2020). And in the specific case of patient retention and adherence indicators during the therapeutic process, EHRs are likely to provide a more accurate view of the therapeutic reality than ad hoc research (Dziadkowiec et al., 2020; Wiessing et al., 2018).

With this in mind, and considering the evidence to date, the present study aims to identify the predictive capacity of retention and adherence concerning the achievement of therapeutic objectives. The specific objectives proposed are: i) to analyze the relationship between adherence and retention, as quantitative and dichotomous variables, and the type of discharge; ii) to analyze the predictive capacity of retention and adherence, as quantitative and dichotomous variables, for therapeutic discharge; iii) to identify the cut‐offs of retention and adherence that achieve the best balance between sensitivity and specificity concerning therapeutic discharge.

## 2 MATERIALS AND METHODS

### 2.1 Design

#### 2.1.1 Retrospective observational study

##### Participants

We accessed outpatients admitted for the first time for treatment for dependence or harmful use of alcohol, cocaine, cannabis, or opiates, in one of the 121 outpatient treatment centers of the Public Network for Addiction Care in Andalusia (Spain). Patients initiated treatment between January 01, 2015 and December 31, 2016, and each of them was followed during 2 years from treatment initiation. In this period, 13,463 outpatients were admitted to treatment. Of these, 1331 patients were excluded since they were discharged beyond the 2‐year follow‐up period and, therefore, information on their therapeutic success/failure was not available. Also excluded were 175 patients referred to other addiction intervention centers and 50 patients who died during the study period. Thus, the total sample of patients for the analyses was 11,907. Comparison of the sociodemographic and consumption‐related characteristics of excluded patients and treatment patients showed no significant effect sizes to conclude that there are differences between these groups (effect sizes estimated by Cramer's V ranging from *V* = 0.014 to *V* = 0.064).

Most patients were male (83.2%) with a mean age of 34.89 years (SD = 13.29) at the time of admission to treatment. Most patients had completed primary education (58.7%) or secondary education (21.3%). Regarding employment status, 32.5% of the patients were employed, 45.3% were unemployed, 12.4% were studying, 7.0% were retired, and 2.7% were in an unknown employment situation.

According to ICD‐10 criteria, 41.3% of patients were diagnosed with alcohol dependence or harmful use, 29.9% with cocaine, 36.2% with cannabis, and 5.2% with opiates. Excluding tobacco addiction, 47.7% of these patients were diagnosed with dependence or harmful use of more than one drug, and 7.5% of the patients were receiving treatment in other mental health services due to the presence of comorbid mental disorders.

##### Procedure

The data used in the present study belong to the EHR of the Information System of the Andalusian Plan on Drugs. This registry was launched in 2003 and contains patients' clinical history.

The EHR begins with recording information corresponding to the variables of the Treatment Demand Indicator (TDI) Standard Protocol 3.0 of the European Monitoring Center for Drugs and Drug Addiction (EMCDDA, 2012). The TDI provides basic information at the start of treatment on sociodemographic variables, drug use history, previous treatments, and infectious diseases. In addition, clinical data collected during the periodic appointments that patients attend (physicians, psychologists, nurses, and social workers) are incorporated into the clinical history of each patient. In these appointments, each team member (physicians, psychologists, nurses, and social workers) incorporates the relevant information for the treatment of the patients in the EHR. This information includes the diagnosis of the patients according to ICD‐10 criteria, prescribed pharmacological treatment, psychological evaluation and treatments, results of toxicological tests, social status of the patient, and evolution of treatment. All this information is stored in a centralized database, and therapists can access the information at any time. Previous research conducted with this same data set have shown adequate values of reliability (Dacosta‐Sánchez et al., 2021).

In the event that a patient changes addiction centers, the team of the new treatment center asks the patients and the therapeutic team of the old treatment center for authorization to consult their EHR. This limits the possibility of a patient being duplicated in the database, which is common to all public and subsidized centers of the addiction treatment network in Andalusia.

##### Ethics and approvals

The storage and encoding of this data comply with the General Health Law of April 25, 1986 (Spain) and Law 41/2002 of November 14 on patient autonomy, rights, and obligations regarding clinical information and documentation. It also complies with the Organic Law March 2018 of December 5, 2018, on protecting personal data and guaranteeing digital rights, with adaptation to European regulations.

To access the EHRs, the researchers made a request to the General Secretary of Social Services of the Department of Equality and Social Policies of the Regional Government of Andalusia (Spain). This agency provided the principal investigator with the fully anonymized database.

The Research Ethics Committee of the Andalusian Ministry of Health certified the compliance with the necessary requirements for the ethical handling of the information.

##### Measures

***Type of discharge*.** Four levels of this variable have been distinguished, with two levels corresponding to therapeutic discharge and two levels to dropout. The two levels of therapeutic discharge correspond to patients who achieved the therapeutic objectives (therapeutic success), and a distinction was made between: a) therapeutic/non‐readmission: patients who during the two subsequent years did not need further professional addiction support; b) therapeutic/readmission: patients who in the 2 years following therapeutic discharge, had relapsed, and needed the support of the clinical team. The two levels of patients who either dropped out of treatment against clinical judgment, or stopped attending planned appointments without notice, are as follows: c) dropout/readmission: patients who, after missing the last appointment for more than 6 months, reapplied for an appointment for treatment of their addiction within the 2‐year follow‐up; and d) dropout/non‐readmission: patients who dropped out of treatment and did not contact the addiction centers again during the 2‐year follow‐up.

For those patients with readmissions to treatment, the time in treatment after the first dropout or therapeutic discharge was not included in analysis.

***Retention*.** This variable was coded as the number of months in treatment from the first appointment to the last appointment of the patients (either therapeutic discharge or the appointment before dropout). In addition to using the number of months as a quantitative variable, this variable was also dichotomized establishing cut‐offs of 3 and 6 months (Zhang et al., 2003).

***Adherence*.** Following previous studies (Austin et al., 2015; Milward et al., 2014; Raes et al., 2011), this variable was analyzed in the following ways: 1) number of sessions: quantitative variable in terms of number of sessions attended by patients from the start of treatment until discharge from treatment (or dropout); 2) proportion of attendance: quantitative variable calculated as the proportion of scheduled appointments attended. For this purpose, a ratio of attendance to scheduled appointments was calculated. In this case, a value of 1 indicates that the patient had attended 100% of the scheduled appointments; and, 3) proportion of attendance (≥6 appointments): quantitative variable calculated as the proportion of attendance to scheduled appointments, but only for patients who had attended a minimum of six appointments, using this value as an international reference for minimally adequate treatment (Degenhardt et al., 2017).

This variable has also been transformed into a dichotomous variable, following the criteria of previous studies that use attendance to six, eight, and 12 appointments as cut‐offs (Degenhardt et al., 2017; Raes et al., 2011).

##### Analysis

Univariate and bivariate descriptive statistics were used to characterize the sample. Analysis of variance, Pearson's Chi‐square, and *t‐student* tests were used to establish the associations between the variables, indicating the effect sizes as appropriate (Eta‐squared, Cramér's V, and Cohen's d). Effect sizes have been interpreted according to the guidelines of Cohen (1988) and Cramér (1946).

Multinomial logistic regression analyses, adjusted for gender and age, were applied to determine the predictive capacity of retention and adherence for patient discharge. Five models were carried out for quantitative variables, and another five models for dichotomized variables according to the cutoff points determined by previous research (Degenhardt et al., 2017; Raes et al., 2011; Zhang et al., 2003).

Receiver operating characteristic (ROC) analyses were performed to study the cut‐offs of retention and adherence for therapeutic/non‐readmission discharge. Subsequently, these cut‐offs were used to analyze the predictive capacity of adherence and retention (as dichotomous variables).

To examine the validity of our results, a subsample of 50% of patients was randomly selected. In both cases (multinomial logistic regression and ROC analysis) results were replicated (supplementary tables).

The software used for the analyses was STATA V.14 and Statistical Package for Social Sciences V.25.

## 3 RESULTS

### 3.1 Association between discharge type, retention, and adherence

The mean time in treatment was 7.11 (SD = 5.58; median = 6 months), the mean number of sessions attended was 10.5 (SD = 22.0), and the median was five. The mean proportion of appointments attended was 0.71 (SD = 0.24), with a median of 0.75. The semi‐interquartile range of months in treatment was 8 (P25 = 2 and P75 = 10), that of the number of sessions attended was also 8 (P25 = 2 and P75 = 10), while the proportion of appointments attended was 0.36 (P25 = 0.56 and P75 = 0.92).

18.7% of the patients achieved the therapeutic objectives without the need to initiate new treatments, while 1.0% reached the therapeutic objectives but were readmitted within 2 years. 12.4% dropped out of treatment and were subsequently readmitted in the following 2 years, while 67.8% dropped out of treatment and had no contact with the treatment centers in the subsequent 2 years.

Table 1 shows the association between retention and adherence and the types of discharge. It is observed that patients with therapeutic discharges who do not require readmission have higher retention in treatment, attended more therapeutic sessions, and showed a higher proportion of attendance. For the dichotomous variables, it was also observed that the highest retention and adherence corresponded to patients who successfully completed treatment. All the relationships analyzed are statistically significant. The analysis of the effect sizes for the quantitative variables shows that retention and attendance ratios have a moderate/high effect size. In contrast, the number of sessions has a small effect size.

**TABLE 1:** Bivariate analysis of the relationship between therapeutic success, retention, and adherence

	Therapeutic/non‐readmission	Therapeutic/readmission	Dropout/readmission	Dropout/non‐readmission	Statistic (*d*.*f*.)	*p*	Effect size
Quantitative variables (M(SD))							
Retention (months)	10.9 (5.57)	8.68 (4.78)	5.12 (4.53)	6.39 (5.27)	*F* (3, 11,905) = 528.6	0.000	*η* ^2^ = 0.12
Number of sessions	18.5 (33.1)	13.6 (19.6)	5.73 (7.97)	9.12 (19.2)	*F* (3, 11,905) = 137.7	0.000	*η* ^2^ = 0.03
Percentage of attendance	0.87 (0.24)	0.84 (0.25)	0.71 (0.29)	0.67 (0.24)	*F* (3, 11,905) = 440.2	0.000	*η* ^2^ = 0.10
*Number of sessions (*n* = 5717)	23.7 (37.1)	17.6 (21.8)	12.5 (10.9)	17.7 (26.8)	*F* (3, 5716) = 24.69	0.000	*η* ^2^ = 0.01
*Percentage of attendance (*n* = 5717)	0.87 (0.14)	0.84 (0.14)	0.78 (0.18)	0.73 (0.19)	*F* (3, 5716) = 221.7	0.000	*η* ^2^ = 0.10
Dichotomous variables (%)							
≥3 months retention	96.7	89.3	60.5	68.5	*Chi2* (3) = 854.5	0.000	*V* = 0.27
≥6 months retention	83.7	73.6	43.2	52.7	*Chi2* (3) = 840.3	0.000	*V* = 0.26
≥6 attended sessions	73.9	71.9	33.1	43.3	*Chi2* (3) = 831.5	0.000	*V* = 0.26
≥8 attended sessions	56.4	49.6	21.6	31.6	*Chi2* (3) = 617.1	0.000	*V* = 0.23
≥12 attended sessions	34.9	27.3	11.0	18.5	*Chi2* (3) = 385.8	0.000	*V* = 0.18

*Note*: *d*.*f*.: degrees of freedom; readm.: readmitted; M.: mean; *SD*.: standard deviation; *: data analysis excluded patients that attended less than six sessions.

When transforming the quantitative variables into dichotomous variables, the retention cut‐off points at 3 and 6 months provide moderate effect sizes in their association with the type of discharge. Concerning the number of sessions, the 6, 8, and 12 appointment thresholds also show moderate effect sizes, with the six‐session cut‐off showing a slightly greater effect.

### 3.2 Predictive capacity of retention and adherence for therapeutic discharge

Table 2 shows five multinomial logistic regression for the quantitative variables of retention and adherence adjusted by gender and age (Models Q1‐Q5). The relative risk ratio analysis, using the dropout/readmissions patients as a reference, show that longer time treatment, attending more sessions or having a higher proportion of attendance is associated with achieving therapeutic discharges. When PseudoR2, Akaike information criterion (AIC) and Bayesiann information criterion (BIC) are compared between models, the variable “proportion of attendance” appears to be the one that better predicts the type of discharge. However, the values for this variable are only slightly better than those observed for “months in treatment”.

**TABLE 2:** Multinomial logistic regression adjusted for age and gender (base outcome = dropout/readmission)

	RRR	Std. Err.	Z	95% Conf. Interval
Model Q1: Months in treatment (*n* = 11,907): LR Chi2 (9) = 1430.33; *p* = 0.000; PseudoR2 = 0.068; AIC: 19,622.04; BIC: 19,710.66				
Dropout/non‐readmission	1.056	0.007	8.83**	1.044–1.069
Therapeutic/readmission	1.141	0.019	7.79**	1.103–1.179
Therapeutic/non‐readmission	1.218	0.009	27.88**	1.202–1.236
Model Q2: Number of sessions (*n* = 11,907): LR Chi2 (9) = 469.20; *p* = 0.000; PseudoR2 = 0.022; AIC: 20,583.17; BIC: 20,671.79				
Dropout/non‐readmission	1.042	0.005	8.76**	1.032–1.051
Therapeutic/readmission	1.052	0.006	9.00**	1.040–1.064
Therapeutic/non‐readmission	1.056	0.005	11.74**	1.047–1.066
Model Q3: Percentage of attendance (*n* = 11,907): LR Chi2 (9) = 1532.20; *p* = 0.000; PseudoR2 = 0.070; AIC: 19,578.99; BIC: 19,667.71				
Dropout/non‐readmission	0.962	0.05	−6.63**	0.951–0.973
Therapeutic/readmission	1.167	0.03	5.93**	1.109–1.228
Therapeutic/non‐readmission	1.207	0.010	21.94	1.187–1.228
Model Q4: Number of sessions (patients who attend ≥6 sessions; *n* = 5717): LR Chi2 (9) = 101.22; *p* = 0.000; PseudoR2 = 0.010; AIC: 10,599.91; BIC: 10,679.73				
Dropout/non‐readmission	1.018	0.004	4.35**	1.010–1.026
Therapeutic/readmission	1.018	0.006	3.06**	1.006–1.030
Therapeutic/non‐readmission	1.024	0.004	5.75**	1.016–1.002
Model Q5: Percentage of attendance (patients who attend ≥6 sessions; *n* = 5717): LR Chi2 (9) = 718; *p* = 0.000; PseudoR2 = 0.067; AIC: 9983.13; BIC: 10,062.95				
Dropout/non‐readmission	0.921	0.012	−6.05**	0.897–0.946
Therapeutic/readmission	1.125	0.044	3.04**	1.043–1.213
Therapeutic/non‐readmission	1.182	0.018	10.51**	1.146–1.219

*Note*: AIC, Akaike Information Criterion; BIC, Bayesian Information Criterion; RRR: Relative Risk‐Ratio; Std. Err.: standard error; Conf.: confidence; *p < 0.05; **p < 0.01.

Table 3 shows multinomial logistic regressions for variables “months in treatment” and “number of sessions” dichotomized (Models D1‐D5). Relative risk ratio values show that these dichotomous variables are also associated with therapeutic discharges (therapeutic/readmissions and therapeutic/non‐readmissions). Moreover, among the dichotomous models, when a cut‐off point of 3 months in treatment is used for dichotomous models, an improve in the prediction of type of discharge is observed. However, PseudoR2, AIC and BIC values suggest that the fit of these models is worse than the fit of the models including quantitative variables.

**TABLE 3:** Multinomial logistic regression adjusted for age and gender (base outcome = dropout/readmission) for dichotomous independent variables

	RRR	Std. Err.	z	95% Conf. Interval
Model D1: Patients with equal/more than 3 months in treatment: LR Chi2 (9) = 1168.06; *p* = 0.000; PseudoR2 = 0.056; AIC: 19,884.31; BIC: 19,972.93				
Dropout/non‐readmission	1.413	0.082	5.93**	1.26–1.584
Therapeutic/readmission	5.431	1.620	5.67**	3.026–9.747
Therapeutic/non‐readmission	19.305	2.517	22.71**	14.952–24.925
Model D2: Patients with equal/more than 6 months in treatment: LR Chi2 (9) = 970.73; *p* = 0.000; PseudoR2 = 0.046; AIC: 20,081.63; 20,170.25				
Dropout/non‐readmission	1.460	0.083	6.64**	1.306–1.632
Therapeutic/readmission	3.673	0.781	6.11**	2.421–5.574
Therapeutic/non‐readmission	6.766	0.526	24.60**	5.81–7.88
Model D3: Patients with equal/more than 6 sessions attended: LR Chi2 (9) = 915.41; *p* = 0.000; PseudoR2 = 0.044; AIC: 20,136.95; BIC: 20,225.57				
Dropout/non‐readmission	1.478	0.088	6.57**	1.315–1.662
Therapeutic/readmission	4.734	0.978	7.53**	3.157–7.097
Therapeutic/non‐readmission	5.638	0.413	23.61**	4.883–6.508
Model D4: Patients with equal/more than 8 sessions attended: LR Chi2 (9) = 668.30; *p* = 0.000; PseudoR2 = 0.032; AIC: 20,384.06; BIC: 20,472.68				
Dropout/non‐readmission	1.578	0.106	6.79**	1.383–1.800
Therapeutic/readmission	3.401	0.655	6.35**	2.33–4.963
Therapeutic/non‐readmission	4.614	0.351	20.12**	3.976–5.356
Model D5: Patients with equal/more than 12 sessions attended: LR Chi2 (9) = 415.13; *p* = 0.000; PseudoR2 = 0.020; AIC: 2637.23; BIC: 20,725.85				
Dropout/non‐readmission	1.718	0.149	6.24**	1.449–2.036
Therapeutic/readmission	2.829	0.628	4.68**	1.831–4.372
Therapeutic/non‐readmission	4.168	0.389	15.31**	3.472–5.004

*Note*: AIC, Akaike Information Criterion; BIC, Bayesian Information Criterion; RRR: Relative Risk‐Ratio; Std. Err.: standard error; Conf.: confidence; *p < 0.05; **p < 0.01.

Tables S1 to S4 in Supplementary material replicate the previous results adjusting for patients with dual pathology attending mental health centers, and educational level. These tables show that no significant differences were found when adjusting for age and gender. Moreover, Tables S5 and S6 in Supplementary material replicate the above statistical analyses with 50% of the sample.

### 3.3 Cut‐off for months of retention, number of sessions, and proportion of appointments attended concerning therapeutic discharge

**FIGURE 1:**
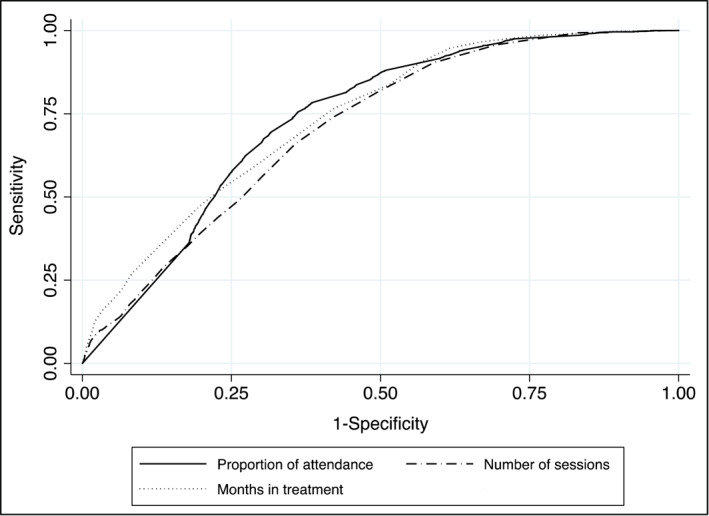
Areas under Receiver Operating Characteristic curves of retention and adherence

Figure 1 shows the ROC curves for the months of retention, frequency of appointments, and proportion of attendance to scheduled appointments. The area under the curve obtained for these three variables is statistically significant, with a value of 0.734 (95% CI = 0.724–0.744) for the proportion of attendance to scheduled sessions, a value of 0.738 (95% CI = 0.728 – 0.748) for months in treatment and 0.708 (95% CI = 0.697–0.718) for the number of sessions attended. In addition, significant differences are observed between the three areas under the curve (Chi2 = 52.73; *p* = 0.000). In the case of proportion of attendance, it is observed that the balance between sensitivity and specificity is achieved with a proportion of. 83 (Sensitivity: 66.34%; Specificity: 69.91%). For months in treatment, the greatest balance between sensitivity and specificity occurs at 8 months (Sensitivity: 66.07%; Specificity: 65.83%). The analysis of the number of sessions shows that the balance is at seven appointments (Sensitivity: 65.98%; Specificity: 64.24%).

## 4 Discussion/Conclusion

The general objective of the present study was to determine the predictive capacity of retention and adherence for therapeutic discharge. Previous studies have focused on the utility of these variables for predicting the therapeutic success of patients in treatment for addiction (Joe et al., 1999). However, to our knowledge, this is the first study that tests the usefulness of these variables jointly and distinguishing them as quantitative or dichotomous variables. In addition, the patients' EHRs have been used, thus maximizing the ecological validity of the data obtained.

Concerning our first objective, it was found that the quantitative variables of retention and adherence are significantly associated with therapeutic success/dropout. However, when using month in treatment and the proportion of attendance to scheduled appointments, the effect size is greater than that observed for adherence estimated by the number of sessions attended. In this sense, although it is common to use of a “minimally adequate” number of sessions to achieve patient improvement in the psychotherapeutic setting (Degenhardt et al., 2017), in our study this variable has a smaller effect on therapeutic success. This discrepancy is possibly due to the fact that in the present study the number of sessions was analyzed without setting a specific period. Therefore, the recommendation of the number of minimally adequate sessions should be accompanied by a temporary period of dispersion. In this regard, some authors suggest that to achieve therapeutic success, it is preferable to schedule the sessions in shorter time intervals rather than spread them over a longer time period (Rawson et al., 2021; Reardon et al., 2002).

Further, transforming the quantitative variables of retention and the number of appointments into dichotomous variables led to a reduction in the effect size observed for retention. However, the effect size slightly increased when using the variable of the number of appointments attended. The transformation of quantitative variables into dichotomous variables may imply a loss of information (Burgette & Paddock, 2017) or statistical power (Shentu & Xie, 2010; Tueller et al., 2016) and induce biases in the interpretation of the results and cause the effects of the treatments to be underestimated in meta‐analyses (DeCoster et al., 2009; Hunter & Schmidt, 1990). However, as Shentu and Xie (2010) point out, this transformation can also lead to a reduction in the error associated with quantitative measurements. It may also be easier to use dichotomous variables for decision‐making with patients in certain clinical contexts. Therefore, the authors believe that the decision to use quantitative or dichotomous variables should be guided by careful consideration of the cases being studied each investigation. It should be the clinicians and researchers who, in each context, should take stock of the benefits and drawbacks of their decisions.

Concerning our second objective, the most striking result to emerge is that the model including the proportion of attendance to appointments as an independent variable better predicts discharge than months in treatment and sessions. It should also be noted that, when transforming the months in treatment variable into a dichotomous variable, the fit decreases significantly for three and 6 months, but placing the cut‐off at 3 months provided very strong fit compared to placing it at 6 months. This result is in accord with the suggestions of other authors regarding the fact that a longer retention time is not always related to patient improvement (Walker, 2009).

In relation with the third objective, statistically significant cut‐offs have been found for variables months in treatment, number of sessions and proportion of attendance. Nevertheless, the sensitivity and specificity values are below what is recommended (Power et al., 2013). Thus, the choice to adopt a specific value as a cut‐off point for these variables should be based on careful consideration on how it may affect each research objective or clinical decision (Trevethan, 2017). On the other hand, although our results and various authors advise against dichotomizing quantitative variables (Fedorov et al., 2009), this is sometimes done to communicate and interpret scientific results more easily, as well as to facilitate clinical decision‐making (DeCoster et al., 2009). Thus, it might be appropriate to provide statistical information for quantitative variables and dichotomous variables when communicating research results. This would benefit those clinicians and researchers with greater statistical skills and would be especially useful for the execution of meta‐analysis studies (Ofuya et al., 2014). Furthermore, depending on the distributions of the variables, cut‐off points consistent with other scientific investigations should be used, which would facilitate the comparability of results between studies.

We consider the results presented to be useful for both researchers and clinicians, highlighting the strength of the sample size used, the validity of the information analyzed, and the impact of the proportion of attendance on therapeutic discharge. This variable, which has been scarcely used to date, may provide the best indicator of the active patient's commitment to his/her treatment (Joe et al., 1999). A further possible advantage of this variable is that it considers retention as both the time elapsed between the first and last scheduled appointment and the number of sessions attended during that time. This is congruent with the claims of various authors, who point out that retention should be studied together with session attendance because it offers more information on the therapeutic process (Pulford et al., 2010; Viera et al., 2020). Furthermore, considering the effect sizes observed for the variables analyzed, we consider it important to complement the use of these indicators with the measurement of other clinical variables and the patients' quality of life.

With respect to the limitations of this study, it is important to consider several methodological issues. For instance, while the statistical analyses controlled for gender and age, certain variables such as the severity of dependency, the sociodemographic profile of the patients (e.g., educational level), and the presence of comorbid mental disorders could also affect the type of discharge (Madoz‐Gúrpide et al., 2013). The inclusion of these variables in the models would probably allow us to achieve greater explanatory capacity. However, including these variables would also produce less parsimonious models that capture the impact of the target variables of this study (retention, appointment attendance, and proportion of appointments attended). On the other hand, and as discussed in the Method section, we excluded 1331 patients who exceeded 2 years of follow‐up. The authors were unable to determine the reasons underlying the extension of treatment beyond 2 years. Therefore, it is difficult to hypothesize the impact of their exclusion on the observed outcomes. However, the meta‐analysis conducted by Beaulieu et al. (2021) on the efficacy of long‐term treatment in substance use disorder indicates that after 18 months, treatment efficacy is often similar in terms of abstinence or moderate drug use. From this perspective, the indicator of time in treatment could be less useful than the percentage attendance at appointments. However, it is necessary to keep in mind that for certain patients, SUD treatment should be equivalent to that offered for chronic diseases, and therefore, only by offering treatment without time limits can patients be expected to make progress in their dependence and improve their quality of life (McLellan et al., 2005).

Finally, our sample contained a notably lower percentage of women than men. Therefore, although the data analyzed here are from patients who have attended treatment, caution should be exercised in generalizing the results to women. Future studies could specifically analyze the variables studied here and their relationship with gender.

## AUTHOR CONTRIBUTIONS

Fermin Fernández‐Calderón, Óscar M. Lozano and Manuel Sánchez‐García have been implicated in the study design.

Daniel Dacosta, Bella González, Manuel Sánchez‐García and Óscar M. Lozano have been involved in data analysis.

All authors contributed to the drafting and revision of the manuscript.

## CONFLICT OF INTEREST

The author declares that there is no conflict of interest that could be perceived as prejudicing the impartiality of the research reported.

## ETHICS STATEMENT

The storage and encoding of this data comply with the General Health Law of April 25, 1986 (Spain) and Law 41/2002 of November 14 on patient autonomy, rights, and obligations regarding clinical information and documentation. It also complies with the Organic Law March 2018 of December 5, 2018, on protecting personal data and guaranteeing digital rights, with adaptation to European regulations.

To access the EHRs, the researchers made a request to the General Secretary of Social Services of the Department of Equality and Social Policies of the Regional Government of Andalusia (Spain). This agency provided the principal investigator with the fully anonymized database.

The Research Ethics Committee of the Andalusian Ministry of Health certified the compliance with the necessary requirements for the ethical handling of the information.

## Supporting information

Supporting Information S1

## Data Availability

The data that support the findings of this study are available from the corresponding author upon reasonable request.
