# Supplementary material for: Retention in treatment and therapeutic adherence: How are these associated with therapeutic success? An analysis using real‐world data
Source: Int J Methods Psychiatr Res. 2022 Jun 28;31(4):e1929. doi: 10.1002/mpr.1929 (PMC9720222; doi:10.1002/mpr.1929)
Supplement: Supplementary file 1 — Supporting Information S1 [file MPR-31-e1929-s001.docx]

**Table S1.** Multinomial logistic regression adjusted by age, gender and comorbidities (base outcome = dropout/readmission)

|  | RRR | Std. Err. | z | 95% Conf. Interval |
| --- | --- | --- | --- | --- |
| Model Q1: Months in treatment (n=11907): LR Chi2(12) = 1512.54; p = .000; PseudoR2 = .071 | | | | |
| Dropout/non-readmission | 1.059 | 0.007 | 9.32** | 1.047 – 1.073 |
| Therapeutic/readmission | 1.140 | 0.019 | 7.70** | 1.102 – 1.177 |
| Therapeutic/non-readmission | 1.224 | 0.009 | 28.33** | 1.207 – 1.242 |
| Model Q2: Number of sessions (n=11907): LR Chi2(12) = 537.01; p = .000; PseudoR2 = .025 | | | | |
| Dropout/non-readmission | 1.045 | 0.005 | 9.21** | 1.035 – 1.055 |
| Therapeutic/readmission | 1.056 | 0.006 | 9.36** | 1.044 – 1.068 |
| Therapeutic/non-readmission | 1.060 | 0.005 | 12.07** | 1.050 – 1.070 |
| Model Q3: Proportion of attendance (n=11907): LR Chi2(9) =1526.32; p = .000; PseudoR2 = .073 | | | | |
| Dropout/non-readmission | 0.962 | 0.010 | -6.60** | 0.951 – 0.973 |
| Therapeutic/readmission | 1.171 | 0.031 | 5.98** | 1.112 – 1.233 |
| Therapeutic/non-readmission | 1.207 | 0.010 | 21.82** | 1.187 – 1.227 |
| Model Q4: Number of sessions (patients who attend ≥ 6 sessions; n=5717): LR Chi2(12) = 133.97; p = .000; PseudoR2 = .013 | | | | |
| Dropout/non-readmission | 1.020 | 0.004 | 4.58** | 1.011 – 1.029 |
| Therapeutic/readmission | 1.021 | 0.006 | 3.41** | 1.009 – 1.033 |
| Therapeutic/non-readmission | 1.026 | 0.005 | 5.85** | 1.018 – 1.035 |
| Model Q5: Proportion of attendance (patients who attend ≥ 6 sessions; n=5717): LR Chi2(9) = 738.03; p = .000; PseudoR2 = .069 | | | | |
| Dropout/non-readmission | 0.922 | 0.013 | -5.95** | 0.898 – 0.947 |
| Therapeutic/readmission | 1.139 | 0.045 | 3.27** | 1.054 – 1.232 |
| Therapeutic/non-readmission | 1.182 | 0.019 | 10.49** | 1.145 – 1.219 |

Note. RRR: Relative Risk-Ratio; Std. Err.: standard error; Conf.: confidence; *p<.05; ** p<.01

**Table S2.** Multinomial logistic regression adjusted by age, gender and comorbidities (base outcome = dropout/readmission)

for dichotomous independent variables

|  | RRR | Std. Err. | z | 95% Conf. Interval |
| --- | --- | --- | --- | --- |
| Model D1: Patients with equal/more than 3 months in treatment: LR Chi2(9) =1233.03; p = .000; PseudoR2 = .058 | | | | |
| Dropout/non-readmission | 1.446 | 0.085 | 6.30** | 1.289 – 1.623 |
| Therapeutic/readmission | 5.391 | 1.610 | 5.64** | 3.002 – 9.681 |
| Therapeutic/non-readmission | 19.857 | 2.592 | 22.89** | 15.374 – 25.647 |
| Model D2: Patients with equal/more than 6 months in treatment: LR Chi2(9) = 1034.8; p = .000; PseudoR2 = .049 | | | | |
| Dropout/non-readmission | 1.488 | 0.085 | 6.95** | 1.330 – 1.665 |
| Therapeutic/readmission | 3.648 | 0.777 | 6.07** | 2.402 – 5.539 |
| Therapeutic/non-readmission | 6.924 | 0.540 | 24.81** | 5.942 – 8.067 |
| Model D3: Patients with equal/more than 6 sessions attended: LR Chi2(12) =993.29; p = .000; PseudoR2 = .047 | | | | |
| Dropout/non-readmission | 1.546 | 0.085 | 7.24** | 1.374 – 1.740 |
| Therapeutic/readmission | 4.730 | 0.982 | 7.48** | 3.148 – 7.107 |
| Therapeutic/non-readmission | 5.971 | 0.442 | 24.16** | 5.165 – 6.902 |
| Model D4: Patients with equal/more than 8 sessions attended: LR Chi2(12) = 741.91; p = .000; PseudoR2 = .035 | | | | |
| Dropout/non-readmission | 1.646 | 0.111 | 7.36** | 1.441 – 1.880 |
| Therapeutic/readmission | 3.379 | 0.656 | 6.27** | 2.310 – 4.945 |
| Therapeutic/non-readmission | 4.863 | 0.373 | 20.63** | 4.185 – 5.652 |
| Model D5: Patients with equal/more than 12 sessions attended: LR Chi2(12) = 485.05; p = .000; PseudoR2 = .023 | | | | |
| Dropout/non-readmission | 1.800 | 0.157 | 6.73** | 1.517 – 2.136 |
| Therapeutic/readmission | 2.790 | 0.624 | 4.59** | 1.800 – 4.324 |
| Therapeutic/non-readmission | 4.413 | 0.415 | 15.79** | 3.671 – 5.306 |

Note. RRR: Relative Risk-Ratio; Std. Err.: standard error; Conf.: confidence; *p<.05; ** p<.01

**Table S3.** Multinomial logistic regression adjusted by age, gender and educational level (base outcome = dropout/readmission)

|  | RRR | Std. Err. | z | 95% Conf. Interval |
| --- | --- | --- | --- | --- |
| Model Q1: Months in treatment (n=11907): LR Chi2(21) = 1576.65; p = .000; PseudoR2 = .075 | | | | |
| Dropout/non-readmission | 1.056 | 0.007 | 8.82** | 1.043 – 1.069 |
| Therapeutic/readmission | 1.141 | 0.019 | 7.79** | 1.103 – 1.179 |
| Therapeutic/non-readmission | 1.221 | 0.009 | 27.99** | 1.204 – 1.238 |
| Model Q2: Number of sessions (n=11907): LR Chi2(21) = 593.34; p = .000; PseudoR2 = .028 | | | | |
| Dropout/non-readmission | 1.041 | 0.005 | 8.71** | 1.032 – 1.051 |
| Therapeutic/readmission | 1.051 | 0.006 | 8.86** | 1.040 – 1.063 |
| Therapeutic/non-readmission | 1.056 | 0.005 | 11.57** | 1.046 – 1.066 |
| Model Q3: Proportion of attendance (n=11907): LR Chi2(21) =1586.26; p = .000; PseudoR2 = .075 | | | | |
| Dropout/non-readmission | 0.962 | 0.010 | -6.65** | 0.950 – 0.973 |
| Therapeutic/readmission | 1.166 | 0.030 | 5.90** | 1.108 – 1.227 |
| Therapeutic/non-readmission | 1.206 | 0.010 | 21.69** | 1.186 – 1.227 |
| Model Q4: Number of sessions (patients who attend ≥ 6 sessions; n=5717): LR Chi2(21) = 181.07; p = .000; PseudoR2 = .017 | | | | |
| Dropout/non-readmission | 1.020 | 0.004 | 4.61** | 1.011 – 1.029 |
| Therapeutic/readmission | 1.020 | 0.006 | 3.32** | 1.008 – 1.032 |
| Therapeutic/non-readmission | 1.026 | 0.005 | 5.86** | 1.017 – 1.035 |
| Model Q5: Proportion of attendance (patients who attend ≥ 6 sessions; n=5717): LR Chi2(21) = 774.39; p = .000; PseudoR2 = .073 | | | | |
| Dropout/non-readmission | 0.923 | 0.012 | -5.91** | 0.899 – 0.948 |
| Therapeutic/readmission | 1.136 | 0.045 | 3.23** | 1.051 – 1.228 |
| Therapeutic/non-readmission | 1.181 | 0.019 | 10.47** | 1.145 – 1.218 |

Note. RRR: Relative Risk-Ratio; Std. Err.: standard error; Conf.: confidence; *p<.05; ** p<.01

**Table S4.** Multinomial logistic regression adjusted by age, gender and educational level (base outcome = dropout/readmission)

for dichotomous independent variables

|  | RRR | Std. Err. | z | 95% Conf. Interval |
| --- | --- | --- | --- | --- |
| Model D1: Patients with equal/more than 3 months in treatment: LR Chi2(21) =1287.97; p = .000; PseudoR2 = .061 | | | | |
| Dropout/non-readmission | 1.412 | 0.082 | 5.92** | 1.260 – 1.584 |
| Therapeutic/readmission | 5.380 | 1.606 | 5.64** | 3.001 – 9.657 |
| Therapeutic/non-readmission | 19.015 | 2.481 | 22.57** | 14.724 – 24.555 |
| Model D2: Patients with equal/more than 6 months in treatment: LR Chi2(21) =1111.91; p = .000; PseudoR2 = .053 | | | | |
| Dropout/non-readmission | 1.461 | 0.082 | 6.65** | 1.307 – 1.634 |
| Therapeutic/readmission | 3.683 | 0.784 | 6.12** | 2.427 – 5.589 |
| Therapeutic/non-readmission | 6.812 | 0.531 | 24.61** | 5.847 – 7.937 |
| Model D3: Patients with equal/more than 6 sessions attended: LR Chi2(21) =1021.29; p = .000; PseudoR2 = .048 | | | | |
| Dropout/non-readmission | 1.479 | 0.088 | 6.56** | 1.316 – 1.662 |
| Therapeutic/readmission | 4.667 | 0.965 | 7.45** | 3.112 – 6.998 |
| Therapeutic/non-readmission | 5.499 | 0.404 | 23.21** | 4.761 – 6.350 |
| Model D4: Patients with equal/more than 8 sessions attended: LR Chi2(21) = 792.41; p = .000; PseudoR2 = .038 | | | | |
| Dropout/non-readmission | 1.580 | 0.106 | 6.81** | 1.385 – 1.802 |
| Therapeutic/readmission | 3.365 | 0.650 | 6.29** | 2.305 – 4.913 |
| Therapeutic/non-readmission | 4.556 | 0.347 | 19.90** | 3.924 – 5.290 |
| Model D5: Patients with equal/more than 12 sessions attended: LR Chi2(21) = 543.29; p = .000; PseudoR2 = .026 | | | | |
| Dropout/non-readmission | 1.720 | 0.149 | 6.24** | 1.450 – 2.038 |
| Therapeutic/readmission | 2.789 | 0.620 | 4.61** | 1.804 – 4.311 |
| Therapeutic/non-readmission | 4.091 | 0.382 | 15.07** | 3.406 – 4.913 |

Note. RRR: Relative Risk-Ratio; Std. Err.: standard error; Conf.: confidence; *p<.05; ** p<.01

**Table S5** Multinomial logistic regression adjusted by age and gender (base outcome = dropout/readmission) (50% of sample)

|  | RRR | Std. Err. | z | 95% Conf. Interval |
| --- | --- | --- | --- | --- |
| Model Q1: Months in retention: LR Chi2(9) =709.36; p = .000; PseudoR2 = .068; AIC: 9733.16; BIC: 9813.37 | | | | |
| Dropout/non-readmission | 1.054 | 0.009 | 6.14** | 1.037 – 1.073 |
| Therapeutic/readmission | 1.104 | 0.026 | 4.11** | 1.053 – 1.157 |
| Therapeutic/non-readmission | 1.220 | 0.012 | 19.76** | 1.197 – 1.244 |
| Model Q2: Number of sessions: LR Chi2(9) =242.31; p = .000; PseudoR2 = .023; AIC: 10200.21; BIC: 10280.42 | | | | |
| Dropout/non-readmission | 1.038 | 0.006 | 6.03** | 1.026 – 1.051 |
| Therapeutic/readmission | 1.048 | 0.008 | 5.99** | 1.032 – 1.064 |
| Therapeutic/non-readmission | 1.054 | 0.007 | 8.39** | 1.041 – 1.067 |
| Model Q3: Proportion of attendance: LR Chi2(9) =694.05 ; p = .000; PseudoR2 = .067; AIC: 9692.92; BIC: 9773.04 | | | | |
| Dropout/non-readmission | 0.962 | 0.008 | -4.50** | 0.946 – 0.978 |
| Therapeutic/readmissions | 1.197 | 0.044 | 4.89** | 1.114 – 1.287 |
| Therapeutic/non-readmission | 1.203 | 0.014 | 14.96** | 1.175 – 1.233 |
| Model Q4: Number of sessions (patients who attend ≥ 6 sessions; n=2824): LR Chi2(9) = 55.16; p = .; PseudoR2 = .010; AIC: 5242.76; BIC: 5314.11 | | | | |
| Dropout/non-readmission | 1.017 | 0.005 | 3.06** | 1.006 – 1.028 |
| Therapeutic/readmission | 1.016 | 0.006 | 2.03* | 1.001 – 1.033 |
| Therapeutic/non-readmission | 1.023 | 0.006 | 4.13** | 1.012 – 1.035 |
| Model Q5: Proportion of attendance (patients who attend ≥ 6 sessions; n=2824): LR Chi2(9) = 325; p = .000; PseudoR2 = .062; AIC: 4954.27; BIC: 5025.56 | | | | |
| Dropout/non-readmission | 0.923 | 0.018 | -4.02** | 0.887 – 0.959 |
| Therapeutic/readmission | 1.142 | 0.064 | 2.37* | 1.023 – 1.275 |
| Therapeutic/non-readmission | 1.176 | 0.027 | 7.01** | 1.124 – 1.231 |

Note. RRR: Relative Risk-Ratio; Std. Err.: standard error; Conf.: confidence; *p<.05; ** p<.01

**Table S6** Multinomial logistic regression adjusted by age and gender (base outcome = dropout/readmission)

for dichotomous independent variables (50% of sample)

|  | RRR | Std. Err. | z | 95% Conf. Interval |
| --- | --- | --- | --- | --- |
| Model D1: Patients with equal/more than 3 months in treatment: LR Chi2(9) =584.14; p = .000; PseudoR2 = .056; AIC: 9858.38; BIC: 9938.59 | | | | |
| dropout/non-readmission | 1.351 | 0.111 | 3.65** | 1.1469 – 1.588 |
| therapeutic/readmission | 4.098 | 1.500 | 3.85** | 1.999 – 8.400 |
| therapeutic/non-readmission | 20.539 | 3.998 | 15.53** | 14.025 – 30.079 |
| Model D2: Patients with equal/more than 6 months in treatment: LR Chi2(9) =517.93; p = .000; PseudoR2 = .050; AIC: 9924.59; BIC: 10004.8 | | | | |
| dropout/non-readmission | 1.499 | 0.121 | 5.04** | 1.281 – 1.756 |
| therapeutic/readmission | 2.537 | 0.680 | 3.48** | 1.501 – 4.290 |
| therapeutic/non-readmission | 7.792 | 0.890 | 17.97** | 6.229 – 9.747 |
| Model D3: Patients with equal/more than 6 sessions attended: LR Chi2(9) =476.11; p = .000; PseudoR2 = .046; AIC: 9966.40; BIC: 10046.62 | | | | |
| dropout/non-readmission | 1.477 | 0.124 | 4.64** | 1.253 – 1.743 |
| therapeutic/readmission | 3.671 | 0.979 | 4.88** | 2.177 – 6.192 |
| therapeutic/non-readmission | 5.993 | 0.630 | 17.03** | 4.877 – 7.365 |
| Model D4: Patients with equal/more than 8 sessions attended: LR Chi2(9) = 341.62; p = .000; PseudoR2 = .033; AIC: 10100.9; BIC: 10181.11 | | | | |
| dropout/non-readmission | 1.545 | 0.146 | 4.62** | 1.285 – 1.859 |
| therapeutic/readmission | 2.259 | 0.601 | 3.06** | 1.341 – 3.805 |
| therapeutic/non-readmission | 4.682 | 0.504 | 14.34** | 3.791 – 5.782 |
| Model D5: Patients with equal/more than 12 sessions attended: LR Chi2(9) = 231.31; p = .000; PseudoR2 = .022; AIC: 10211.21; BIC: 10291.42 | | | | |
| dropout/non-readmission | 1.625 | 0.196 | 4.02** | 1.283 – 2.059 |
| therapeutic/readmission | 1.869 | 0.616 | 1.90 | 0.979 – 3.565 |
| therapeutic/non-readmission | 4.278 | 0.558 | 11.13** | 3.312 – 5.526 |

Note. RRR: Relative Risk-Ratio; Std. Err.: standard error; Conf.: confidence; *p<.05; ** p<.01

**Figure S1** Areas under Receiver Operating Characteristic curves of retention and adherence (50% of sample)
